# Piperine ameliorates insulin resistance via inhibiting metabolic inflammation in monosodium glutamate-treated obese mice

**DOI:** 10.1186/s12902-020-00617-1

**Published:** 2020-10-07

**Authors:** Chaolong Liu, Yanting Yuan, Ji Zhou, Ruixin Hu, Lixia Ji, Guohui Jiang

**Affiliations:** School of Pharmacy, Qingdao University, Qingdao, 266021 Shandong China

**Keywords:** Piperine, Obesity, Insulin resistance, Chronic low-grade inflammation

## Abstract

**Background:**

Metabolic inflammation is an essential event in obesity-induced diabetes and insulin resistance. In obesity, an increasing number of macrophages recruited into visceral adipose tissues undergo significant M_1_-like polarization, secreting variable amounts of pro-inflammatory cytokines and causing insulin resistance. Piperine has excellent anti-inflammatory activities and may be used in the treatment of a variety of inflammatory diseases. In this study, we investigated the effect of piperine on adipose tissue inflammation and insulin resistance in obese mice.

**Methods:**

Newborn mice were subcutaneously (s.c.) injected with monosodium glutamate (MSG) to establish a diabetes model. After 24 weeks, the MSG obese mice were divided into three groups and treated with piperine (40 mg/kg/day), metformin (150 mg/kg/day) and vehicle for 10 successive weeks, respectively.

**Results:**

The obesity model was successfully established, as the body weight, insulin resistance, fasting blood glucose (FBG) and dyslipidemia were significantly increased. The 10-week administration of piperine to the obese mice not only significantly decreased the elevated FBG (Model: 6.45 ± 0.41 mM; Piperine: 4.72 ± 0.44 mM, *p* < 0.01), serum TC (Model: 5.66 ± 0.66 mM; Piperine: 3.55 ± 0.30 mM, *p <* 0.01) and TG (Model: 1.41 ± 0.08 mM; Piperine: 0.94 ± 0.05 mM, *p* < 0.001), but also enhanced the glucose infusion rate in the hyperglycemic clamp experiment. Meanwhile, piperine improved glucose intolerance and insulin resistance in MSG obese mice. Piperine markedly decreased the total and differential white blood cell (WBC) count, the serum levels of lipopolysaccharide (LPS) and pro-inflammatory cytokines such as galectin-3 (Gal-3) and interleukin-1β (IL-1β). Furthermore, piperine clearly down-regulated the mRNA levels of pro-inflammatory cytokines and the protein levels of M_1_-like polarization marker CD11c and Gal-3 in adipose tissues. The in vitro study showed that piperine inhibited LPS-stimulated polarization of RAW 264.7 cells toward the M_1_ phenotype.

**Conclusions:**

Piperine served as an immunomodulator for the treatment of obesity-related diabetes through its anti-inflammatory effects, which might be achieved by inhibiting macrophages M_1_ polarization in adipose tissues.

## Background

Obesity and type 2 diabetes mellitus (T2DM) have been a great threat worldwide in recent years. According to the Global Diabetes Atlas (9th edition, International Diabetes Federation), the global incidence of diabetes was 9.3%, and the total number of patients was 463 million in 2019. By 2045, these two figures will increase to 10.9% and 700 million, respectively [1]. Obesity is one of the main causes of metabolic diseases including T2DM, steatohepatitis, fatty liver diseases, and many cardiovascular diseases [2–5]. The reason for this correlation is that both obesity and T2DM are chronic low-grade inflammatory diseases [6, 7]. It is worth noting that macrophages play an essential role in obesity and T2DM. Firstly, the number of macrophages is significantly increased in the adipose tissue of both obese rodents and humans [8]. Further studies showed that the increase of the macrophages is mainly because of the increase of pro-inflammatory macrophages, i.e., the M_1_-polarized macrophages. The ratio of M_1_ to M_2_ macrophages is also increased, which leads to metabolic inflammations in adipose tissues. Chronic tissue inflammation leads to increased levels of pro-inflammatory cytokines, such as TNF-α and IL- lβ, which impair insulin signaling and induce insulin resistance [9, 10]. In addition to the classic pro-inflammatory cytokines mentioned above, Pingping Li et al. found that the inflammatory mediator galectin-3 (gal-3), which is mainly secreted by M_1_-like macrophages in visceral adipose tissues, can inhibits the downstream signaling of the insulin receptor (IR) by directly binding with IR, leading to systemic insulin resistance [11–13]. These pieces of evidence indicate that modulation of the conversion of M_1_ to M_2_-like polarized state of macrophages, either by genetic or pharmacological methods, is a promising approach for the treatment of obesity-induced insulin resistance and diabetes.

In this study, the obese mice were obtained by monosodium glutamate (MSG) neonatal intoxication. The obese mice show hypothalamic lesions, and neuro-endocrine changes have been observed in the insulin and leptin signaling [14]. MSG obese mice are characterized by sloth and more closely resemble human central obesity and T2DM [15, 16]. The mice gradually develop obvious centripetal obesity, oral glucose intolerance, metabolic inflammation and insulin resistance from the 2nd month onwards, and high FBG is observed after 4 months. Adult MSG obese mice have typical properties of obesity associated with insulin resistance and T2DM. Hence, this model is suitable for the investigation of obesity-related metabolic dysfunctions [17, 18].

Piperine is the major alkaloid presented in black pepper (*Piper nigrum*), long pepper (*Piper longum*), and many other piper species. Piperine exhibits a wide range of biological properties, such as immunomodulation, anti-oxidation, anti-lipid metabolism disorder, and anti-inflammation [19–21]. Among these pharmacological activities, what attracts us the most is its excellent modulatory effect on immune-inflammation in disease models such as clone diseases, arthritis and ulcerative colitis [22–24]. Although some previous studies have confirmed the beneficial effect of piperine in improving dyslipidemia and reducing body fat accumulation in HFD-induced mice [25, 26], it is unclear whether piperine can improve metabolic disorders by inhibiting the metabolic inflammation in obesity. Therefore, the aim of this study was to explore the role of piperine and it implications in the treatment of obesity-induced metabolic dysfunction and prevention of related chronic low-grade inflammation.

## Methods

### Animal model establishment and experimental design

**Fig. 1:**
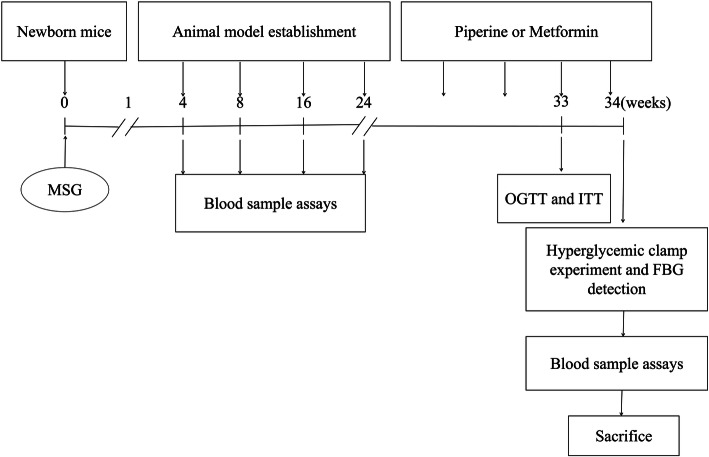
The experimental protocol for studying the effect of piperine on the development of insulin resistance in MSG-obese mice. MSG-obese mice were obtained by s.c. injection of MSG (4 g/kg/d) to newborn mice for 7 successive days. After 24 weeks, the obese MSG mice were divided into three groups and treated with piperine (40 mg/kg/d), metformin (150 mg/kg/d), or vehicle, respectively, for 10 weeks

Pregnant ICR mice were purchased from Vital River Laboratory Animal Technology (Beijing, China). Mice were adapted to laboratory condition for 1 week before experiment. On the day of birth, the newborn mice were randomly divided into two groups. On postnatal day 2, the MSG-treated pups were injected subcutaneously (s.c.) with MSG (4 g/kg/d, Sigma-Aldrich, USA) for 7 consecutive days. Pups in the control group were s.c. injected with an equivalent volume of 0.9% physiological saline solution [16, 27]. Weaning after 21 days, MSG and control mice were separated by gender. Biochemical and physiological determinations were performed in these two groups at 1, 2, 4 and 6 months of age. The mice were allowed free access to water and a chow diet. All mice were kept in the Laboratory Animal Center of Qingdao University at an ambient temperature of 25 ± 2 °C, 12 h light/dark cycles and humidity of 40–60%. After 24 weeks, compared with the control mice, the MSG mice showed significant centripetal obesity, accompanied by elevated serum TC, TG, and insulin levels. Insulin resistance was increased as well. Based on the body weight, Lee’s index, serum TC, TG and FBG, the 6-month-old MSG mice were divided into three groups: (i) the Model group, which was administered an equal volume of saline. (ii) the Piperine group and Metformin group, which were treated with 40 mg piperine/kg/day (Sigma-Aldrich, USA) and 150 mg metformin/kg/day (Sigma-Aldrich, USA) by gavage for 10 weeks, respectively. Meanwhile, the 6-month-old normal mice without MSG treatment were selected as normal controls. There were 8 mice per group. The food intake and body weight of the mice were recorded weekly during the 10-week period of the treatment. The experimental protocols are outlined in Fig. 1. All animal protocols were performed according to the Guidelines for the Care and Use of Laboratory Animals prepared and approved by the Animal Care and Use Committee of the Affiliated Hospital, Qingdao University and the Animal Experimental Ethical Committee of Affiliated Hospital, Qingdao University, Shandong Province, China.

### Measurement of visceral organ indices and the collection of serum

The mice were euthanized by opening the heart under anesthesia with pentobarbital (50 mg/kg, Sigma-Aldrich, USA). Then, the abdominal adipose, pancreas, liver, and kidney were collected and weighted before frozen in liquid nitrogen until further analysis. RNA extraction and real-time PCR analysis were performed using these tissue samples. Blood samples were prepared by centrifuge at 4000 rpm for 10 min at 4 °C. The samples were stored at − 80 °C for later analyses. Throughout the experiments, all mice were provided with housing that allows the expression of species-specific behaviors. Appropriate anesthesia was used to minimize pain, and the mice were trained to cooperate with procedures to minimize any distress. Thus, the experimentally induced stress was minimized. All experimental procedures conformed to the European Guidelines for the care and use of Laboratory Animals (directive 2010/63/EU).

### Oral glucose tolerance test (OGTT)

Seven days prior to the experiments, 4-h-fasted mice received glucose (2 g/kg body weight) by gavage. A total of 3.5 μL blood was collected from the tip of the tail vein at different time points (0, 30, 60, and 120 min) after glucose load, which was used for blood glucose determination with a one Touch Ultra glucose meter (ACCU-CHEK Performa Nano, Roche Diabetes Care GmbH). The area under the curve (AUC) was calculated according to the following formula, AUC = 1/4(PG0min + PG30min) + 1/4(PG30min + PG60min) + 1/2(PG60min + PG120min), where, PG0min, PG30min, PG60min, and PG120min are the blood glucose level at 0, 30, 60, and 120 min after glucose load, respectively.

### Hyperglycemic clamp experiment

At the last week of the experiment, the mice fasted for 4 h were anesthetized with 50 mg/kg (i.p.) of pentobarbital, and the tracheotomy procedure was performed. When the animals were stabilized after 30 min post the operation, the hyperglycemic clamp experiment was performed. First, an initial dose of glucose (250 mg/kg B.W.) was injected through a jugular vein tube to quickly increase blood glucose to a higher level within 5 min, and then a variable rate of glucose (20%, w/v) was infused. The blood glucose levels were measured at 5-min intervals until the blood glucose level reached a steady state (between 13.5 and 14.5 mmol/L). After the steady state, the glucose infusion rate measured at 5 time points was averaged as the glucose infusion rate (GIR) at the steady state.

### Insulin tolerance test (ITT)

The mice were administered with insulin (0.4 unit/kg, novolin, Novo Nordisk, Danmark) 4 h after fasting via intraperitoneal injection. The blood glucose levels were calculated using blood samples collected at 0, 40 and 90 min after insulin injection. The percentage of blood glucose reduction at 40 min was calculated accordingly.

### Routine blood test and biochemical analysis

The blood WBC counts were detected on a PE-6800VET Automated Animal Blood Cell Analyzer (Pukang Inc., Shenzhen, China). Serum total cholesterol (TC) and triglyceride (TG) were measured by an enzymatic colorimetric method using a commercially available kit (Jiancheng Bioengineering Institute, Nanjing, China). Fasting blood glucose was detected by a one Touch Ultra Meter (ACCU-CHEK Performa Nano, Roche Diabetes Care GmbH).

### Enzyme-linked immunosorbent assays

Serum insulin was determined by using a mouse ultrasensitive insulin ELISA kit (American Laboratory Products Company, USA). LPS produced by the Gram-negative bacteria in the intestine, M_1_-like pro-inflammatory cytokines (IL-1β and Gal-3) and M_2_-like anti-inflammatory cytokine (IL-10) in the serum samples were measured by an ELISA kit (Mlbio, Shanghai, China).

### Semiquantitative reverse transcriptase polymerase chain reaction

Total RNA was extracted from the adipose tissues or cultured cells homogenized in Trizol (CWBIO, Beijing, China), which was used for the synthesis of cDNA. qRT-PCR amplification (TaKaRa Bio, Shiga, Japan) was performed on a BioRad CFX96 detection system (BioRad, Hercules, CA, USA) using the primers obtained from the GeneBank. The expression levels of the genes were measured relative to the β-actin level and evaluated using the 2-ΔΔCT method. The primer sequences are presented in Table 1.

**Table 1:** Primer sequences for qRT-PCR

Gene	Primer sequences
CD11c	Forward:5′-ACACAGTGTGCTCCAGTATGA-3′ Reverse:5′-GCCCAGGGATATGTTCACAGC-3′
IL-1β	Forward:5′-AAATACCTGTGGGCCTTGGGC-3′ Reverse:5′-CTGGGATCCACACTCTCCAG-3′
TNF-α	Forward:5′-CCAGACCCTCACACTCAGATC-3′ Reverse:5′-CACTTGGTGGTTTTGCTACGAC-3’
Galectin-3	Forward:5’-TCCTGGAGGCTATCCTGCTG-3′ Reverse:5′-TGTTTGCGTTGGGTTTCACTG-3’
β-actin	Forward:5’-AAGAGAGGCATCCTGACCCT-3′ Reverse:5′-TACATGGCTGGGGTGTTGAA-3’

### Morphologic and immunohistochemical analysis

The abdominal adipose and liver were collected at the end of the experiment, fixed with 4% polyformalin in PBS solution for over 24 h and embedded in paraffin. Hepatic fat lesions and adipocyte diameters in abdominal adipose were observed by HE staining for morphologic analysis. Paraplast-embedded sections (5 μm) were subject to immunoperoxidase staining. The sections were incubated with anti-mouse CD11c (1:250, Affinity, USA) and Galectin-3 (1:250, Abcam, Cambridge, USA) antibody. Amplification and staining were performed using an avidin-biotin-complex and 3,3-diaminobenzidine method, respectively.

### Cell culture and treatment

RAW 264.7 macrophages were obtained from ATCC, USA and cultured in DMEM supplemented with 10% FBS, 100 U/ml penicillin and 100 μg/ml streptomycin (Gibco, USA) in a 5% CO_2_ humidified cell incubator at 37 °C. Piperine was added to the cell culture to a final concentration of 0, 20, 40, and 80 μM, respectively, and the macrophages were incubated for 12 h. LPS (1 μg/mL, Sigma-Aldrich, USA) was added afterward to induce M_1_ macrophage polarization. After the treatment, cell-free supernatants were collected to determine the IL-1β level using an ELISA kit assay, and the cells were collected for RNA as described above. The piperine solution was prepared by dissolving piperine in DMSO. The final concentration of DMSO in the cell culture should not exceed 0.05%.

### Western blot analysis

The stimulated cells were homogenized in RIPA buffer supplemented with protease and phosphatase inhibitors (Slarbio, Beijing, China) after being washed with phosphate buffered saline (PBS) twice. The homogenate was subject to 10–12% SDS-PAGE electrophoresis before transferred to a PVDF membrane, which was then incubated with the primary antibody CD11c, Toll like receptor-4 (TLR-4) (1:1000; Affinity, USA) and IL-1β (1:2000; Abcam Cambridge, MA, USA) overnight at 4 °C, followed by incubation with the HRP-linked secondary antibody at 25 °C for 2 h. An eECL western blot kit (CWBIO, Beijing, China) was used to detect the proteins.

### Statistical analyses

The software GraphPad Prism 7.00 was used for data analyses. All values are reported as means ± SD. An unpaired t-test was used for comparisons between two groups. One-way ANOVA was used for comparisons among three or more groups. *p* < 0.05, *p* < 0.01, *p* < 0.001 and *p* < 0.0001 were used to represent the levels of significant difference.

## Results

### Effects of piperine on body weight, mesenteric fat accumulation, dietary intake and Lee’s index

**Fig. 2:**
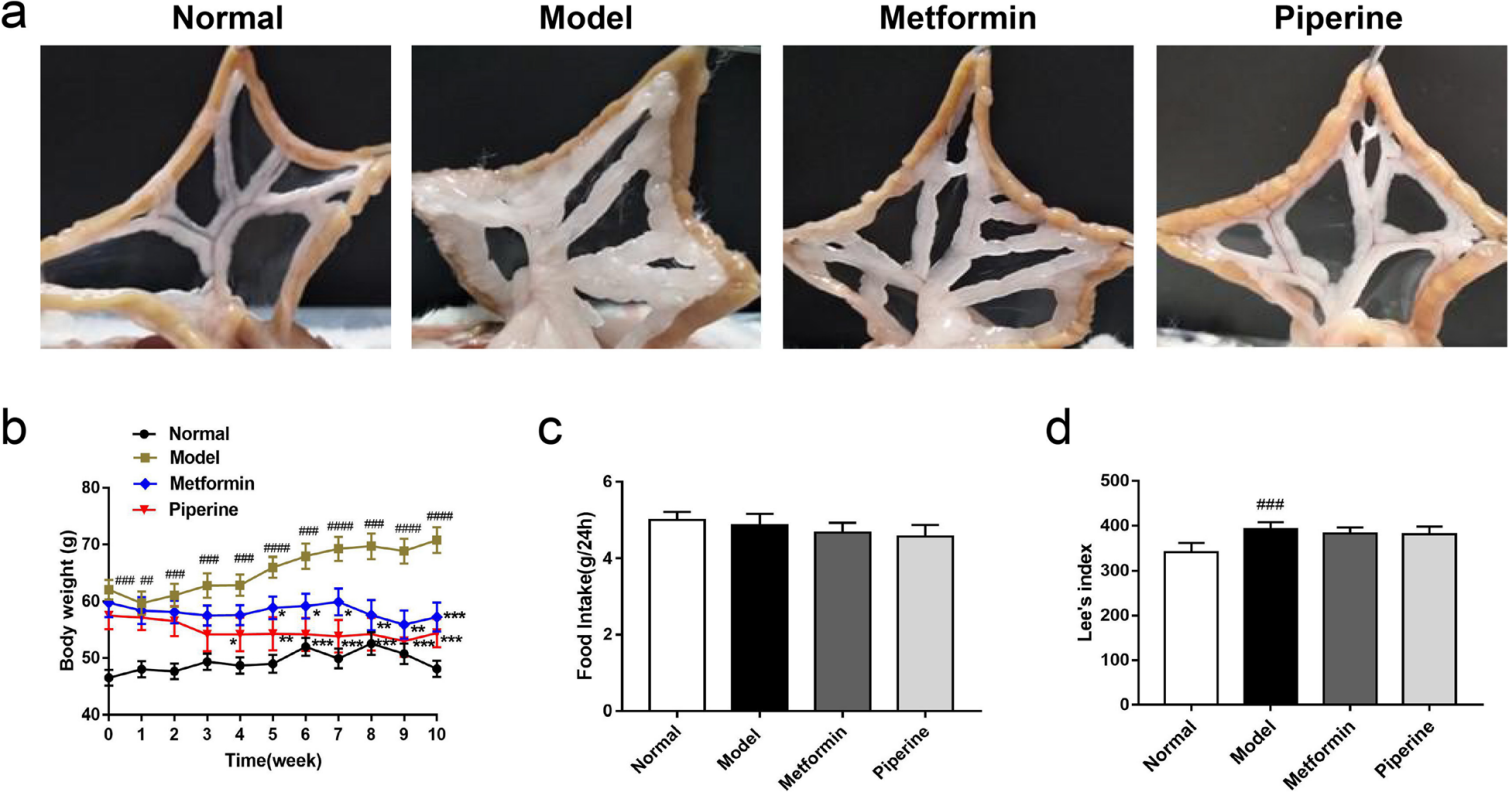
Piperine reduced mesenteric fat accumulation and body weight but not food intake and Lee’s index. **a** Piperine reduced mesenteric fat accumulation. **b** Piperine inhibited the MSG-induced body weight gain. **c**-**d** Piperine did not change the daily food intake and Lee’s index. Data are expressed as mean ± SD, *n* = 6–8. ^##^*p* < 0.01, ^###^*p* < 0.001, ^####^*p* < 0.0001 vs. Normal group; *p < 0.05, **p < 0.01, ***p < 0.001 vs. Model group

To explore the effect of piperine on the established obesity, body weight, mesenteric fat accumulation, Lee’s index, glycolipid metabolism and insulin sensitivity were assessed in the MSG-obese insulin resistant mice upon the piperine treatment. The results showed that MSG caused more mesenteric fat accumulation and body weight gain in MSG-obese insulin resistant mice (Model: 70.20 ± 2.54 g, *p* < 0.0001) than normal mice (Normal: 48.09 ± 1.43 g). In contrast, the piperine treatment relieved mesenteric fat accumulation and body weight gain (Fig. 2a-b). It was found that from the 4th week onwards, the body weight of the piperine-treated mice began to decrease and eventually reached 53.0 ± 2.88 g, significantly lower (*p* < 0.001) than that of the MSG-obese mice (70.20 ± 2.54 g). No significant differences of daily food intake and Lee’s index were observed between the model and piperine-treated groups (*p* > 0.05, Fig. 2c-d).

### Effects of piperine on visceral organ indices

**Fig. 3:**
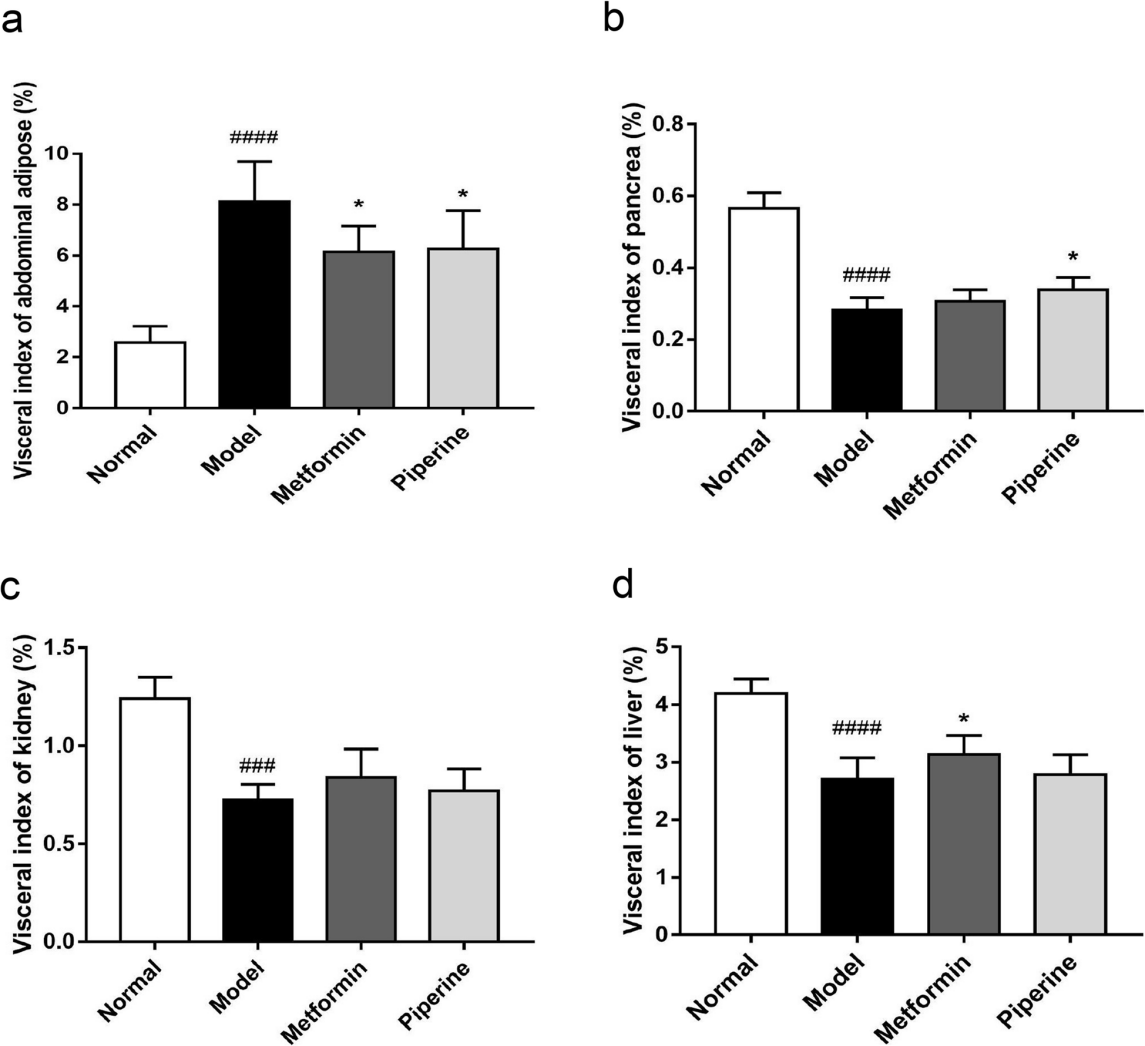
The effect of piperine on visceral organ indices. **a** Piperine decreased the visceral index of abdominal adipose. **b** Piperine increased the visceral index of pancreas. **c**-**d** Piperine did not change the visceral index of kidney and liver. Data are expressed as mean ± SD, *n* = 6–8. ^###^*p* < 0.001, ^####^*p* < 0.0001 vs. Normal group; *p < 0.05 vs. Model group

In order to evaluate the function of organs, the relative weight of the abdominal fat, pancreas, kidney, and liver was calculated after the mice were sacrificed. Compared to the normal group (visceral index of abdominal adipose: 2.58 ± 0.24%; pancreas index: 0.57 ± 0.02%; kidney index: 1.24 ± 0.04%; liver index: 4.20 ± 0.10%), the MSG-obese mice had a higher visceral index of abdominal adipose (8.11 ± 0.56%) and a lower visceral index of pancreas (0.28 ± 0.01%), kidney (0.72 ± 0.03%) and liver (2.71 ± 0.13%). The differences between groups were significant (*p* < 0.001). As expected, the piperine treatment completely reduced the visceral index of the abdominal adipose (6.27 ± 0.61%, *p* < 0.05) (Fig. 3a) and increased the visceral index of the pancreas (0.34 ± 0.01%, *p <* 0.05) in the MSG obese mice (Fig. 3b). The data suggested that piperine might protect the pancreas to a certain extent and restore the relative weight of the pancreas. Besides, we found that the piperine treatment did not change the relative weight of kidney (Fig. 3c) and liver (Fig. 3d) (*p* > 0.05).

### Effects of piperine on the changes of glycolipid metabolism parameters

**Fig. 4:**
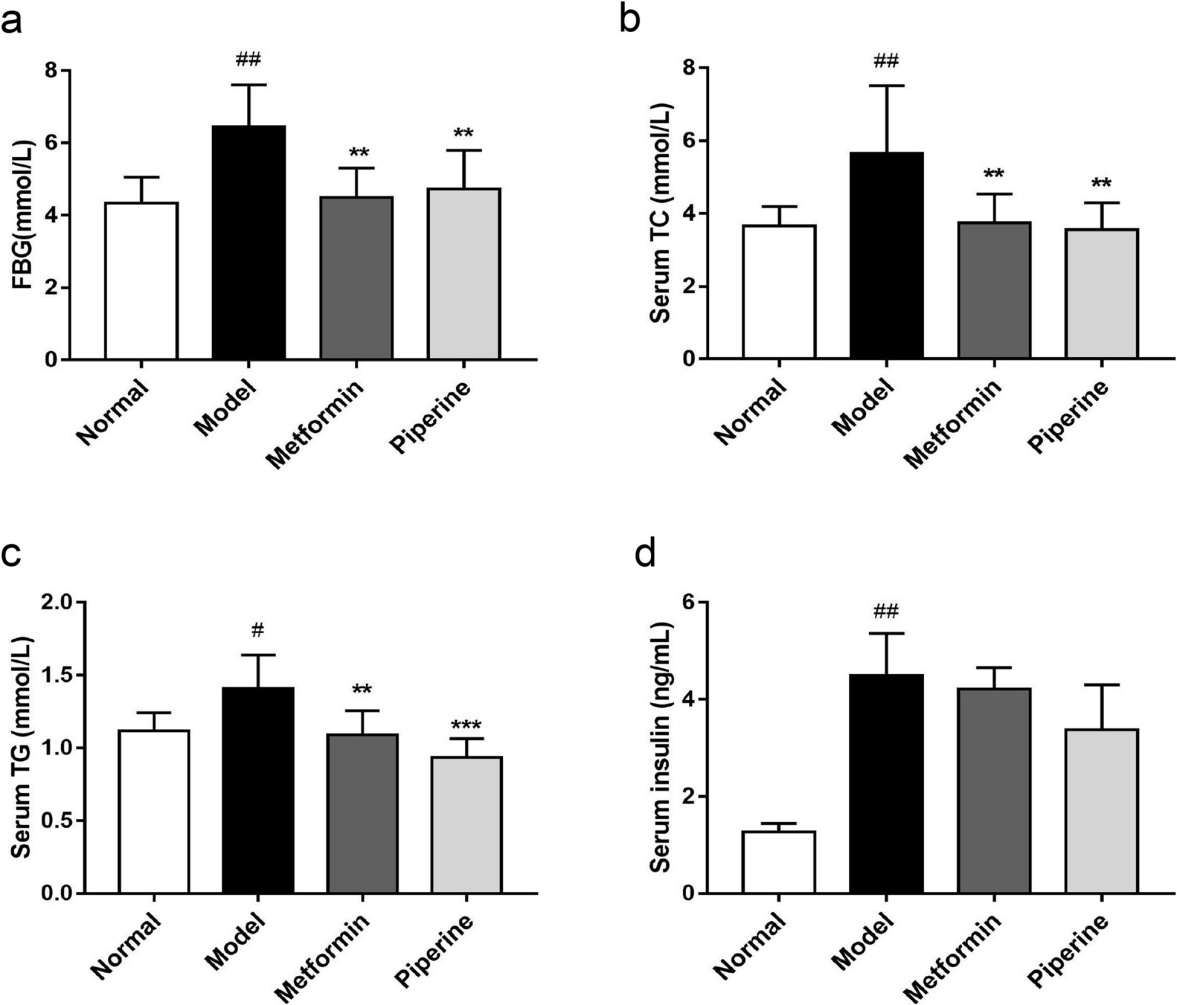
Piperine regulated glycolipid metabolism in MSG mice. **a** Piperine decreased the level of FBG. **b**-**c** Piperine decreased the serum levels of TC and TG. **d** Piperine slightly improved hyperinsulinemia in MSG obese mice. Data are expressed as mean ± SD, *n* = 6–8. ^#^*p* < 0.05, ^##^*p* < 0.01 vs. Normal group; **p < 0.01, ***p < 0.001 vs. Model group

Obesity is one of the main factors causing glycolipid metabolism disorder. The regulatory effect of piperine on glycolipid metabolism was examined in this section. As assumed, the MSG obese mice had higher levels of FBG (Model: 6.45 ± 0.41 mM; Normal: 4.32 ± 0.28 mM, *p <* 0.01), serum TC (Model: 5.66 ± 0.66 mM; Normal:3.67 ± 0.20 mM, *p* < 0.01) and TG (Model: 1.41 ± 0.08 mM; Normal: 1.12 ± 0.05 mM, *p* < 0.05). In contrast, the piperine treatment dramatically reduced the levels of FBG (4.72 ± 0.44 mM, 26.82% reduction compared with the model group, *p <* 0.01), serum TC (3.55 ± 0.30 mM, 37.28% reduction, *p* < 0.01) and TG (0.94 ± 0.05 mM, 33.33% reduction, *p* < 0.001) (Fig. 4a-c). Additionally, the MSG mice showed obvious hyperinsulinemia (Model: 4.50 ± 0.87 ng/mL; Normal: 1.28 ± 0.17 ng/mL, *p <* 0.01). A decreasing trend of the serum insulin level (3.37 ± 0.93 ng/mL, *p* > 0.05) was also observed upon the piperine administration. However, it is not statistically different (Fig. 4d). The results showed that piperine had a similar effect on glucose metabolism compared with metformin, but might have a better effect on lipid metabolism.

### Effects of piperine on oral glucose tolerance test, hyperglycemic clamp experiment and insulin tolerance test

Oral glucose tolerance test (OGTT) and hyperglycemic clamp experiment were used to assess the effect of piperine on glucose tolerance. The effect of piperine on insulin sensitivity was evaluated using the insulin tolerance test (ITT). Administration of MSG led to significant glucose intolerance and insulin resistance in the ICR mice, which were markedly attenuated in the piperine-treated MSG mice.

**Fig. 5:**
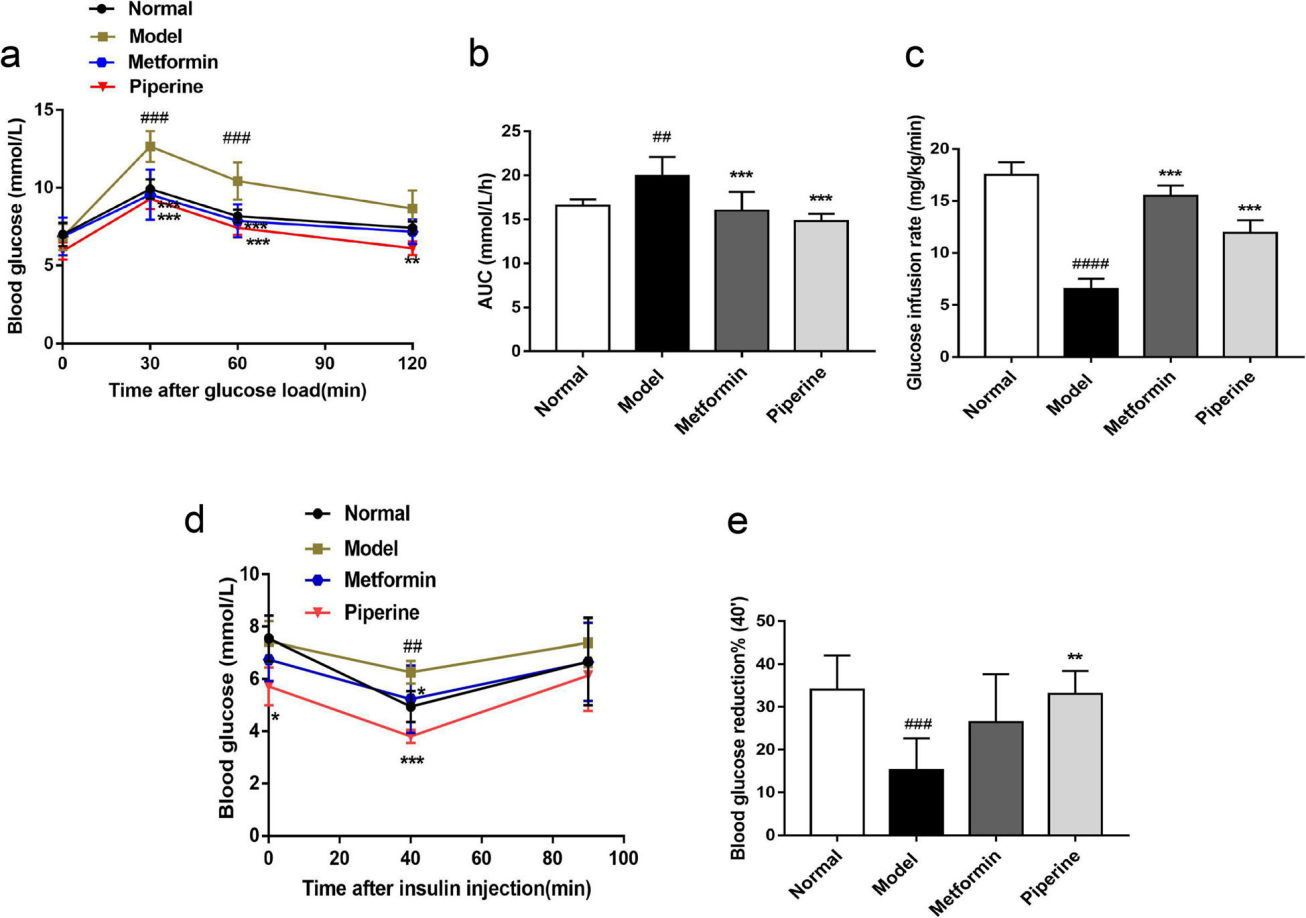
Piperine improved oral glucose intolerance and insulin sensitivity detected by OGTT, hyperglycemic clamp experiment and ITT, respectively. **a** Plasma glucose levels were measured during an OGTT. **b** Area under the curve (AUC) in OGTT. **c** Glucose infusion rate in the hyperglycemic clamp experiment. **d** Plasma glucose levels were measured in ITT. **e** Percentage decrease of blood glucose at 40 min in ITT. Data are expressed as mean ± SD, *n* = 6–8. ^##^*p* < 0.01, ^###^*p* < 0.01 vs. Normal group; *p < 0.05, **p < 0.01,***p < 0.001 vs. Model group

Glucose tolerance results are summarized in Fig. 5a. The results showed that the glucose level in the MSG mice significantly increased in the first 30 min after glucose load (9.91 ± 0.63 mM vs. 12.40 ± 1.46 mM, *p* < 0.001). However, the glucose levels were 25% lower in the piperine treated mice compared with that of the model group (9.28 ± 0.65 mM vs. 12.40 ± 1.46 mM, *p* < 0.001). The results also showed that the integrated glucose level was greatly lowered in the piperine treated mice compared with that of the control mice (Fig. 5b). The glucose level in the MSG mice could be completely recovered by piperine at 40 mg/kg B.W.

The hyperglycemic clamp experiment showed that the GIR of MSG mice was obviously lower than that of the normal group (Model: 6.56 ± 0.39 mg/kg/min; Normal: 17.50 ± 0.51 mg/kg/min, *p* < 0.0001), indicating that there was significant decrease of pancreas function in MSG-obese mice. After 10 weeks of administration, compared with the model group, piperine and metformin increased the GIR of MSG mice by 45.12% (12.01 ± 0.49 mg/kg/min, *p* < 0.001) and 57.61% (15.50 ± 0.41 mg/kg/min, *p <* 0.001), respectively, suggesting that piperine is beneficial to improve the sensitivity of islet β cells to glucose stimulation in obese mice, that is, it can improve the function of islet β cells (Fig. 5c).

The ITT results showed that after 40 min of insulin injection, blood glucose in the piperine group decreased by 33.02%, which is significantly higher than that in the MSG group (15.26%), indicating that the piperine treatment improved systemic insulin sensitivity in the MSG obese mice (Fig. 5d-e).

### Effects of piperine on pathological changes in the liver and abdominal adipose

**Fig. 6:**
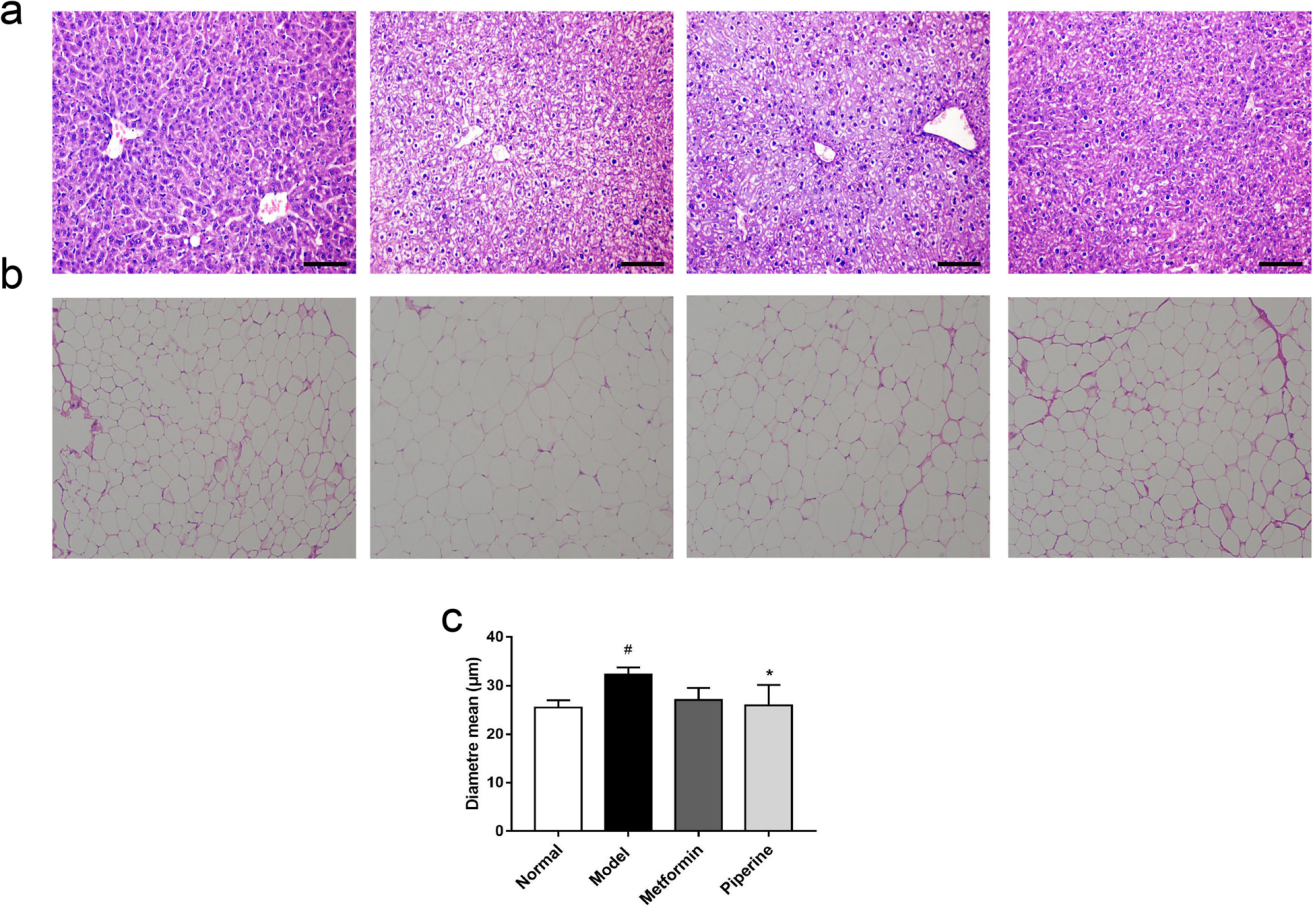
Protective effects of piperine on the liver and adipose tissue morphology. **a** Representative H&E staining of liver tissue sections (200×). The piperine treatment reduced MSG-induced fatty infiltration in liver. **b** Representative H&E staining of abdominal adipose tissue sections (100×). **c** Adipocyte diameters (mean) as measured with H&E staining. The piperine treatment reduced adipocyte diameters. Data are expressed as mean ± SD, *n* = 3. ^#^*p* < 0.05 vs. Normal group; *p < 0.05 vs. Model group

Heavy accumulation of fat in the liver was observed by histomorphological analysis, indicating there were severe pathological changes of nonalcoholic fatty live disease (NAFLD) in the MSG mice. The livers of the model mice showed heavy hepatic steatosis, whereas in the MSG mice, the steatosis was partially relieved by the piperine treatment (Fig. 6a). In some obese patients, insulin resistance occurs due to the accumulation of “dysfunctional” adipose tissues, which are characterized by “large” lipid-laden adipocytes. Our results showed that the adipocyte size was greatly increased in the MSG obese mice, while the hypertrophic adipocyte was ameliorated by the piperine treatment (Fig. 6b-c). These data indicated that piperine played a vital role in regulating lipid metabolism in the abdominal adipose and liver, both of which are the main targets of insulin.

### Effect of piperine on systemic inflammation

**Fig. 7:**
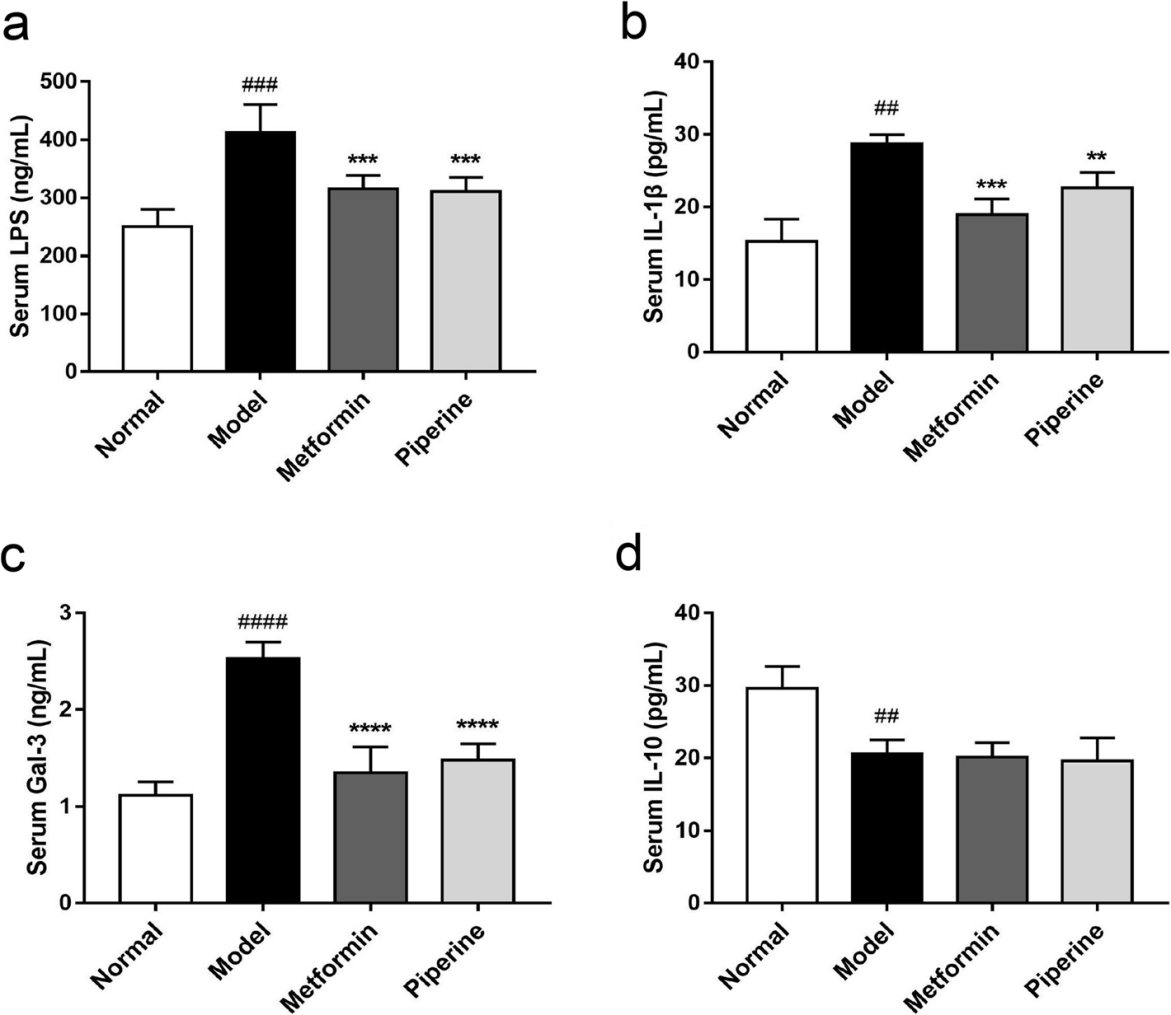
Regulatory effect of piperine on serum inflammatory cytokines in MSG obese mice. **a**-**c** Piperine decreased the levels of serum LPS, IL-1β and Gal-3. **d** Piperine did not change the level of serum IL-10. Data are expressed as mean ± SD, *n* = 6–8. ^##^*p* < 0.01, ^###^*p* < 0.001 vs. Normal group; **p < 0.01, ***p < 0.001 vs. Model group

The routine blood test showed that WBC, lymphocyte, and monocyte in the piperine-treated group were significantly less than those of the model group (Table 2). Besides, we found that endotoxin LPS (Model: 413.20 ± 19.59 ng/mL; Normal: 250.21 ± 12.16 ng/mL, *p* < 0.001), and serum pro-inflammatory cytokines such as IL-1β (Model: 28.78 ± 0.50 pg/mL; Normal: 15.24 ± 1.30 pg/mL, *p* < 0.01) and Gal-3 (Model: 2.53 ± 0.07 ng/mL; Normal: 1.12 ± 0.06 ng/mL, *p* < 0.0001) were significantly elevated in the MSG mice compared with those in the normal group mice. At the end of the 10-week period, the serum levels of LPS (311.20 ± 11.01 ng/mL, *p <* 0.001), IL-1β (22.62 ± 0.88 pg/mL, *p <* 0.01) and Gal-3 (1.48 ± 0.07 ng/mL, *p <* 0.0001) were noticeably reduced in the piperine-treated mice compared with those in the model group mice (Fig. 7a-c). Additionally, although the serum anti-inflammatory cytokine IL-10 in the model mice (20.60 ± 0.78 pg/mL) was much lower than that in the normal mice (29.61 ± 1.35 pg/mL, *p <* 0.01), the administration of piperine did not restore this indicator (19.60 ± 1.29 pg/mL, *p* > 0.05) (Fig. 7d). These results demonstrated that the piperine treatment suppressed the obesity-enhanced inflammatory responses in obese mice.

**Table 2:** Routine blood test in animals

	Normal	Model	Metformin	Piperine
WBC (9 × 10^9^ /L)	5.23 ± 1.02	8.22 ± 1.09^###^	5.73 ± 1.76^**^	4.78 ± 0.98^***^
LYMPH(9 × 10^9^ /L)	3.79 ± 1.26	6.02 ± 1.13^##^	3.87 ± 0.83^**^	3.77 ± 0.53^***^
NEUT(9 × 10^9^ /L)	1.32 ± 0.25	1.26 ± 0.06	1.23 ± 0.13	1.18 ± 0.11
MONO(9 × 10^9^ /L)	0.26 ± 0.10	0.63 ± 0.15^####^	0.23 ± 0.16^****^	0.145 ± 0.04^****^
LYMPH%	68.76 ± 6.75	75.84 ± 3.74	71.97 ± 6.45	71.58 ± 3.35
NEUT%	26.51 ± 6.56	16.18 ± 2.58^##^	23.67 ± 5.45^*^	25.17 ± 2.27^**^
MONO%	4.73 ± 1.81	7.98 ± 1.93^#^	4.36 ± 2.94^*^	3.14 ± 1.12^**^
HGB (g/L)	124.10 ± 16.35	112.82 ± 17.13	118.70 ± 12.81	111.71 ± 8.47
RBC(9 × 10^12^ /L)	8.89 ± 1.21	7.58 ± 1.43	8.14 ± 1.13	7.24 ± 0.44^#^
HCT%	47.46 ± 5.23	42.72 ± 7.04	46.32 ± 3.94	45.02 ± 3.35
MCV (fl)	52.91 ± 2.88	56.83 ± 5.68	57.47 ± 6.64	60.12 ± 4.31^#^
MCH (pg)	13.81 ± 0.50	14.42 ± 0.79	14.63 ± 0.91	15.68 ± 0.61^###^
MCHC(g/L)	261.10 ± 8.01	255.21 ± 21.84	256.20 ± 15.37	245.31 ± 9.16
PLT (fl)	946.11 ± 88.14	889.20 ± 159.51	793.31 ± 131.51	734.22 ± 150.40
MPV (fl)	7.67 ± 0.38	7.88 ± 0.51	7.78 ± 0.79	8.21 ± 1.14

Data are expressed as mean ± SD, *n* = 6–8 per group, ^#^*p* < 0.05; ^##^*p* < 0.01; ^###^*p* < 0.001; ^####^*p* < 0.0001 vs. Normal group; *p < 0.05; **p < 0.01;***p < 0.001;****p < 0.0001 vs. Model group

### Effects of piperine on inflammatory mediators in the adipose tissue

**Fig. 8:**
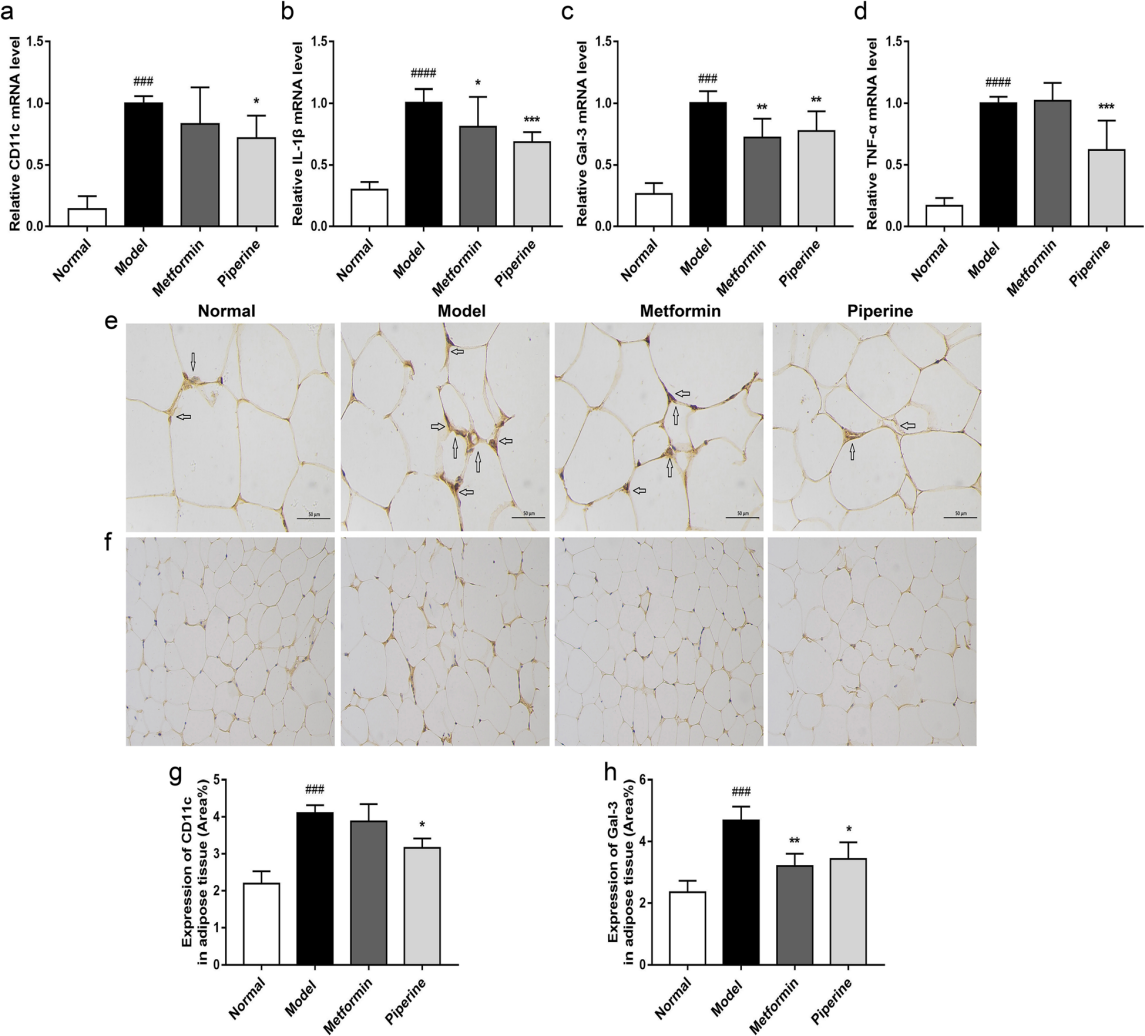
Piperine inhibited the inflammatory state of the adipose tissue in MSG-obese mice. **a** Piperine decreased CD11c mRNA level in WAT. **b**-**d** Piperine reduced IL-1β, Gal-3 and TNF-a mRNA levels in WAT. **e**-**h** The expression of CD11c (400×) and Gal-3 (200×) in abdominal adipose. Data are expressed as mean ± SD, *n =* 6–8. ^##^*p* < 0.01, ^###^*p* < 0.001 vs. Normal group; *p < 0.05, **p < 0.01, ***p < 0.001 vs. Model group

In order to detect the inflammatory status of adipose tissues in each group, the expression of M_1_-like macrophage marker CD11c and related inflammatory cytokines were examined at the mRNA level. qRT-PCR assay showed that the mRNA levels of CD11c, IL-1β, Gal-3 and TNF-a were significantly increased in the adipose tissue in MSG obese mice (*p* < 0.001). In contrast, all these genes were markedly decreased in the piperine-treated group (*p* < 0.05, Fig. 8a-d). The results also demonstrated that IL-1β and Gal-3 were significantly reduced in the metformin-treated group compared to the model group.

We simultaneously measured the protein expression of CD11c and Gal-3 in the visceral adipose tissue. Immunohistochemistry results showed that both CD11c and Gal-3 were over-expressed in the adipose tissue of the MSG group, and the piperine treatment reduced the expression of both CD11c and Gal-3 (Fig. 8e-h). Taken together, these results indicated that piperine alleviated obesity-enhanced M_1_-like polarization of macrophages and inhibited the secretion of pro-inflammatory cytokines in the visceral adipose tissue, which is consistent with the serum levels of pro-inflammatory cytokine. In fact, M_1_-like polarization of macrophages in visceral adipose tissue is the source of systemic inflammation.

### Effect of piperine on in vitro macrophage polarization

**Fig. 9:**
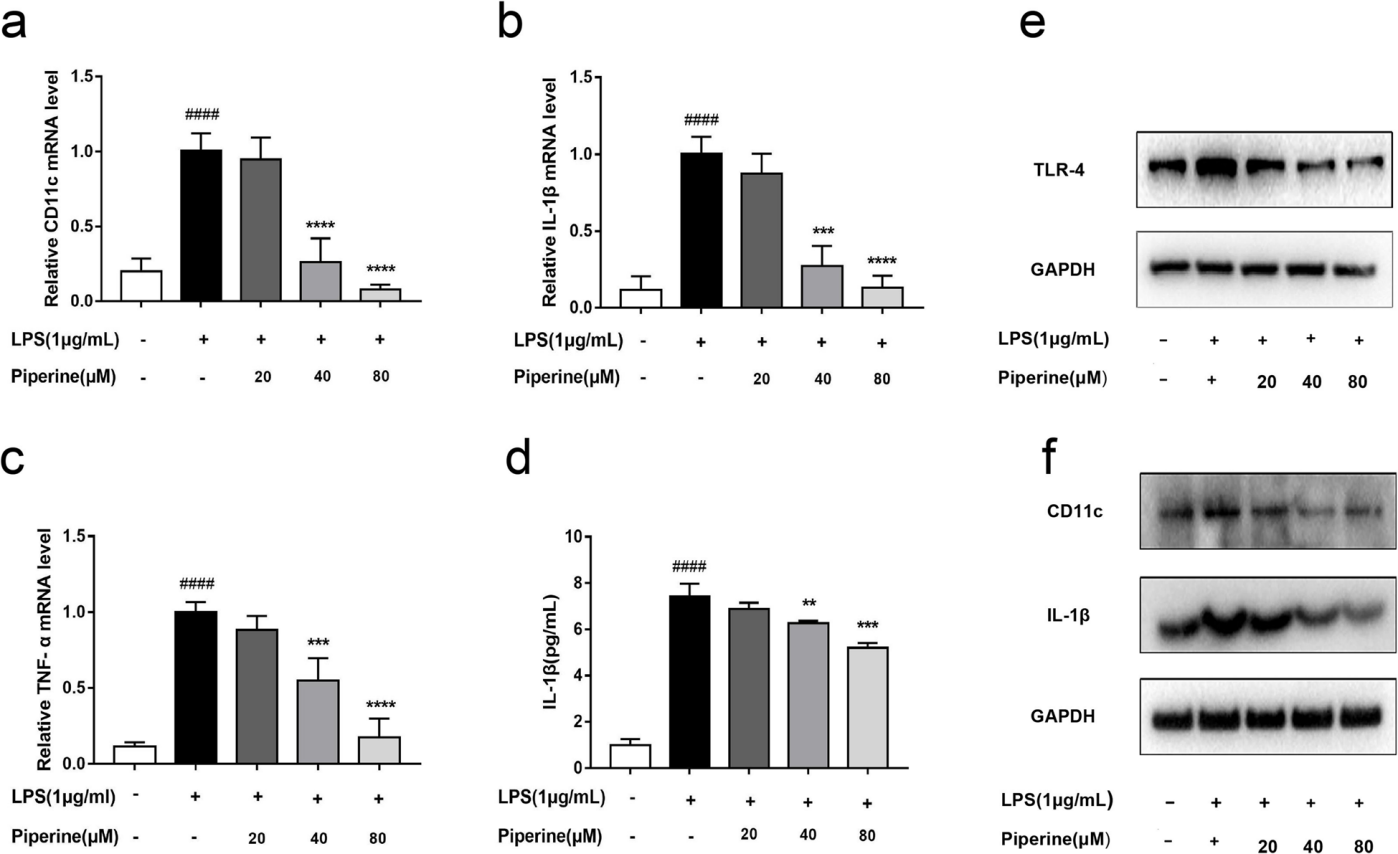
Effects of piperine on LPS-induced M_1_-like polarization in RAW264.7 cells. RAW264.7 cells were pretreated with piperine at 20–80 μM for 12 h and then stimulated by LPS (1 μg/ml) for 24 h. **a**-**c** The mRNA levels of CD11c, IL-1β and TNF-α were analyzed by qPCR. **d** The IL-1β level in cell supernatant was measured by ELISA. **e**-**f** The protein levels of TLR-4, CD11c and IL-1β were tested by Western Blotting. Data are expressed as mean ± SD, *n* = 3. ^####^*p* < 0.0001 vs. Control group; **p < 0.01, ***p < 0.001, ****p < 0.0001 vs. LPS group. (The original, unprocessed gel images are included in the additional file 1)

The inhibitory effect of piperine on M_1_ macrophage polarization was evaluated using an inflammatory cell culture model. As expected, the LPS treatment increased the mRNA expression of TNF-α, IL-1β and CD11c, while piperine inhibited the over-expression of these genes in a concentration-dependent manner in RAW264.7 cells (Fig. 9a-c). ELISA assay showed that LPS-stimulated IL-1β production was also inhibited by piperine (Fig. 9d). The protein levels of TLR-4, CD11c and IL-1β were detected by western blot analysis. The results showed that piperine (20, 40, and 80 μM) inhibited the over-expression of these proteins in the RAW264.7 cells after the LPS treatment (Fig. 9e-f).

## Discussion

In diabetes patients, the severity of insulin resistance is closely associated with the degree of metabolic inflammation [2, 3, 28]. Insulin resistance and T2DM may be cured or alleviated by direct inhibition of inflammatory responses. In this study, we mainly investigated the effect of piperine on obesity-induced metabolic inflammation and insulin resistance. The results showed that the piperine treatment could greatly reduce the obesity of the MSG mice and alleviate the inflammation in adipose tissue macrophages (ATMs), hence protecting MSG obese mice from insulin resistance.

Metformin is a first-line drug recommended in the guidelines for the prevention and treatment of T2DM, so we selected metformin as a positive control drug to compare the therapeutic effect of piperine on obese diabetic mice. The current results indicate that piperine is more potent than metformin in decreasing body weight (24.50% vs. 16.52% reduction compared with the model group) and increasing the relative weight of the pancreas. Furthermore, we observed that piperine decreased hepatic lipid accumulation and plasma content of TC and TG in MSG obese mice, and these effects are better than metformin. It is worth noting that the 40 mg/kg body weight of piperine reduced FBG level and increased insulin sensitivity as effectively as metformin.

Previous studies have shown that insulin resistance and type 2 diabetes (T2D) can be induced by abnormal immune cell activation [29]. Positive correlations between body mass and cell numbers of ATMs indicate that M_1_-polarized macrophages and chronic low-grade inflammation may play a key role in obesity-induced insulin resistance [30]. ATMs undergo not only quantitative increases in adipose tissues, but also qualitative changes in their activated state to aggravate metabolic inflammation during obesity [31]. In healthy/lean adipose tissues, the initially activated macrophages (M_2_-like) are dominant, which only express CD11b and F4/80 on their surface and secrete anti-inflammatory cytokines such as IL-4 and IL-10. These anti-inflammatory cytokines play a key role in maintaining the sensitivity of adipocytes to insulin, thereby inhibiting the lipolysis process [32]. On the contrary, obesity triggers the accumulation of classically activated macrophages (M_1_-like) induced by FFA or LPS. These macrophages tend to over-express a variety of surface markers such as CD11c, CD11b and F4/80, and secrete pro-inflammatory cytokines including IL-6, IL-1β, TNF-α and Gal-3, which impair the insulin signaling pathways [9, 29, 33–35]. In the current study, we found that MSG significantly induced the over-expression of M_1_-like polarization biomarker CD11c at the mRNA and protein levels, which can be greatly alleviated by piperine treatment. In addition, we confirmed the effect of piperine on in vitro macrophage polarzation. The results showed that LPS induced over-expression of CD11c and TLR-4 in macrophages was reduced by the piperine pre-treatment through directly inhibiting the M_1_-like polarization in RAW264.7 cells.

How does the reduced CD11c^+^ ATMs by piperine lead to the improvement of insulin resistance? The main players in this interaction are pro-inflammatory cytokines produced by the inflamed immune cells in visceral adipose tissues. LPS is a strong stimulator to trigger a number of cytokines associated with systemic insulin resistance [36]. Studies have indicated that long-term high-calorie diets change the composition of intestinal microbiota, and the increased number of gram-negative bacteria produced more endotoxin, resulting in a higher plasma level of LPS in obesity patients [37, 38]. LPS binds to the complex of mCD14 and TLR-4 at the surface of the innate immune cells, which activates inflammatory pathways and then triggers the secretion of pro-inflammatory cytokines, thus impacting insulin action [39]. Conversely, TLR-4 over-expression leads to a certain degree of adipose insulin resistance [40–42]. Therefore, the elevation of serum LPS has been considered to be a risk factor of inflammatory disease. Administration of piperine resulted in a reduction in serum level of LPS in MSG obese mice, but the mechanism still unclear. Studies have shown that the restoration of normal sensitivity to insulin is often accompanied by the decrease in inflammatory markers both in the adipose tissue and the systemic level [43]. Hence, to understand whether the improvement of piperine on glycolipid metabolism disorder is associated with metabolic inflammation, we measured the blood total WBC and serum pro-inflammatory cytokine levels. We found that piperine significantly reduced blood total WBC, serum Gal-3 and IL-1β. However, it had no significant effect on the serum level of IL-10. The results indicated that piperine mainly inhibited M_1_-like polarization of macrophages, but had little effect on M_2_-like polarization. Visceral adipose tissue is the original source of metabolic inflammation in obesity. To further confirm our hypothesis, qRT-PCR and immunohistochemistry were performed to detect the expressions of M_1_-like polarization biomarker CD11c and pro-inflammatory cytokines in the visceral adipose tissue. The results show that piperine greatly decreased the mRNA levels of IL-1β, Gal-3 and TNF-α in MSG obese mice. These results confirmed that piperine might be used to improve insulin sensitivity by regulating inflammatory states of macrophages in the visceral adipose tissue.

It has been reported that piperine-supplemented diet can significantly reduce the body and visceral fat by 12 and 38%, respectively, compared with the mice fed with HFD [26]. Choi et al. have shown that the supplement of piperine in a high-fat diet significantly reverses hepatic steatosis and insulin resistance in HFD obese mice [19]. we found that piperine reduced body weight, mesenteric fat accumulation, abdominal adipose index and serum lipid profiles. In addition, the ITT data indicated that piperine could enhance insulin sensitivity and improve insulin resistance in MSG obese mice. Glucose utilization was completely normalized by piperine during the 10-week period, indicating that piperine was beneficial to improve the oral glucose intolerance and the sensitivity of islet β cells to glucose stimulation in obese mice.

Our results demonstrate piperine had a promising role in improving insulin sensitivity and alleviating adipose tissue inflammation in MSG obese mice. The study will pave the path for further studies of the immune-modulatory and anti-inflammatory functions of other alkaloids. However, there are some limitations in the present study. For example, only a single dose of piperine was used. Therefore, we could not explore the effects of piperine at different doses on T2DM. In addition, we only performed the inhibitory effect of piperine on LPS induced macrophage polarization in in vitro experiments, but did not explore the related signaling pathways. The underlying mechanism of piperine needs to be further explored on the regulation of macrophage polarization and the malignant induction between adipocytes and macrophages in obese and T2DM mice models.

## Conclusions

Our study demonstrates that the piperine treatment could lead to moderate body weight loss, significantly reverse glycolipid metabolism disorders, and improve the established insulin resistance and glucose intolerance in MSG-obese mice. The effect of piperine on obesity-associated diabetes is likely to stem from the strong inhibitory effect on systemic and adipose inflammation. Taken together, piperine, as a potential natural alkaloid, shows a great prospect in the development and application of antidiabetic drugs. Further clinical trials need to be conducted to confirm these effects.

## Supplementary information

**Additional file 1: Figure S1.** The original, unprocessed gel images. RAW264.7 cells were pretreated with piperine at 20–80 μM for 12 h and then stimulated by LPS (1 μg/ml) for 24 h. The protein levels of CD11c, IL-1β and TLR-4 were tested by Western Blotting and the original multiple exposure images are included in the Fig. S1.

## Data Availability

We wish not to share the raw data as the authors are aiming for future publications from the data. However, the data used to support the findings of this study are available from the corresponding author upon request.

## Abbreviations

MSG: Monosodium glutamate
B.W: Body weight
TC: Total cholesterol
TG: Total triglycerides
WBC: White blood cell
LPS: Lipopolysaccharide
Gal-3: Galectin-3
IL-1β: Interleukin-1β
IL-4: Interleukin-4
IL-6: Interleukin-6
IL-10: Interleukin-10
T2DM: Type 2 diabetes mellitus
OGTT: Oral glucose tolerance test
GIR: Glucose infusion rate
AUC: Under the curve
PG: Plasma glucose
ITT: Insulin tolerance test
ELISA: Enzyme-linked immunosorbent assays
FBS: Fetal bovine serum
PBS: Phosphate buffered saline
TLR-4: Toll like receptor-4
NAFLD: Nonalcoholic fatty live disease
ATM: Adipose tissue macrophages
WAT: White adipose tissue
TNF-α: Tumor necrosis factor-α
FFA: Free fatty acid
GLUT-4: Glucose transporter-4
HFD: High fat diet
LYMPH: Lymphocyte
NEUT: Neutrophil
MONO: Monocyte
HGB: Hemoglobin
RBC: Red blood cell
HCT: Hematocrit
MCV: Mean corpuscular volume
MCH: Mean corpuscular hemoglobin
MCHC: Mean corpuscular hemoglobin concentration
PLT: Platelets
MPV: Mean platelets volume
