# Supplementary material for: Piperine ameliorates insulin resistance via inhibiting metabolic inflammation in monosodium glutamate-treated obese mice
Source: BMC Endocr Disord. 2020 Oct 7;20:152. doi: 10.1186/s12902-020-00617-1 (PMC7542877; doi:10.1186/s12902-020-00617-1)

Anti-inflammatory effect of piperine ameliorates insulin resistance in monosodium glutamate–treated obese mice

Chaolong Liu[1], Yanting Yuan[1], Ji Zhou[1], Ruixin Hu[1], Lixia Ji[1,*,#], Guohui Jiang[1,*,#]

* Correspondences: Lixia Ji: lixiaji@163.com; Guohui Jiang: 13370830026@163.com

# Lixia Ji and Guohui Jiang contributed equally to this work

1 School of Pharmacy, Qingdao University, Qingdao, Shandong 266021, China

Figure S1. The original, unprocessed gel images

Original multiple exposure CD11c images

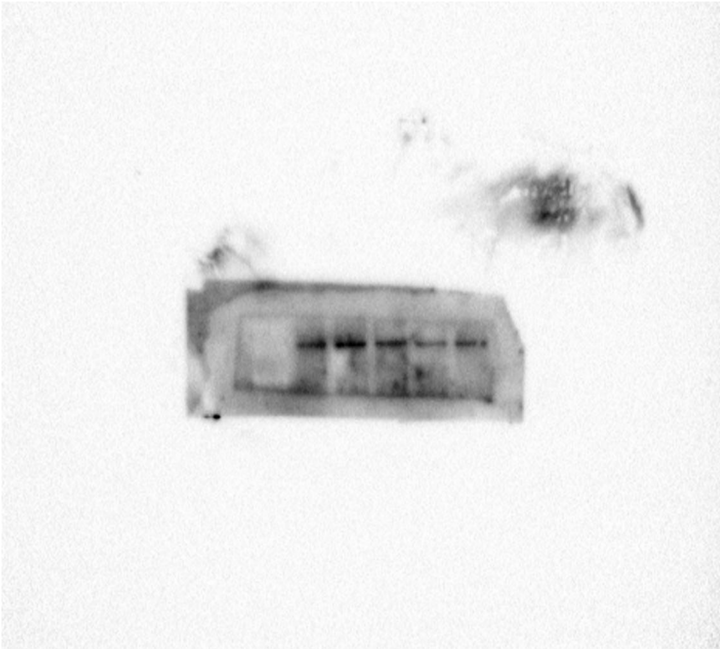

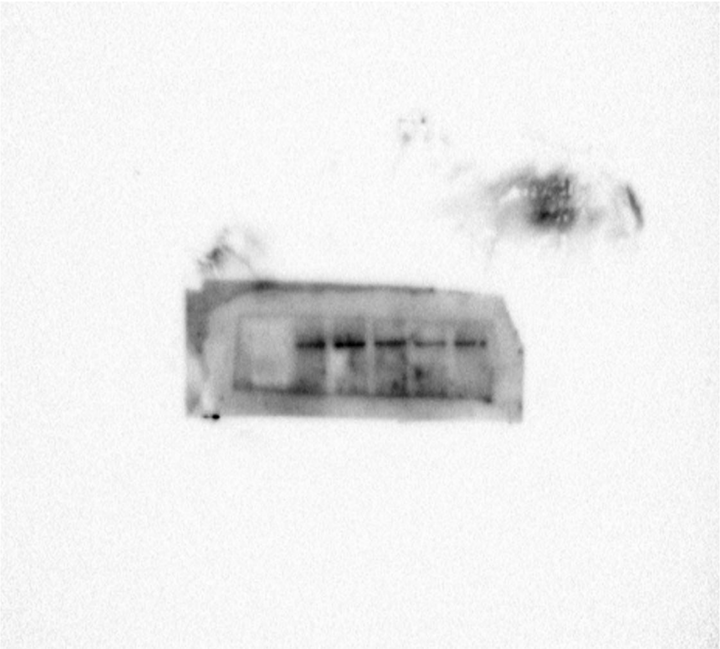

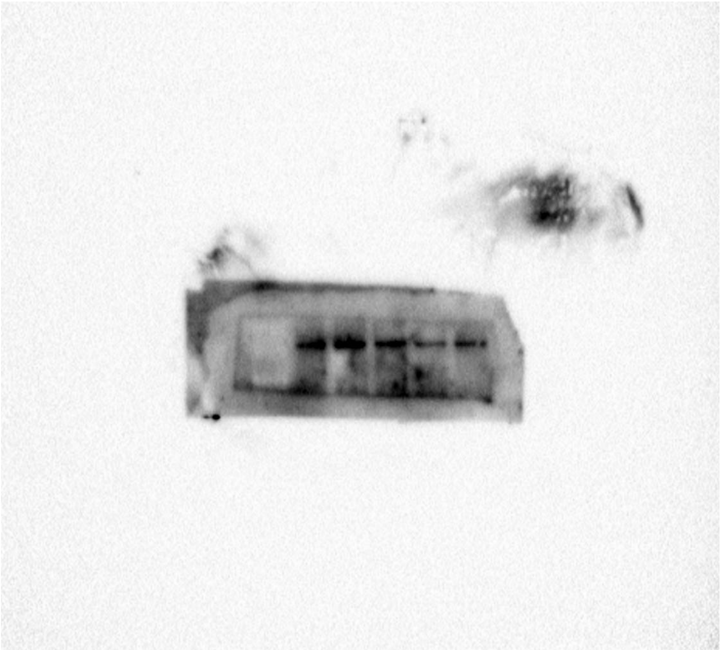

Original multiple exposure GAPDH-1 images

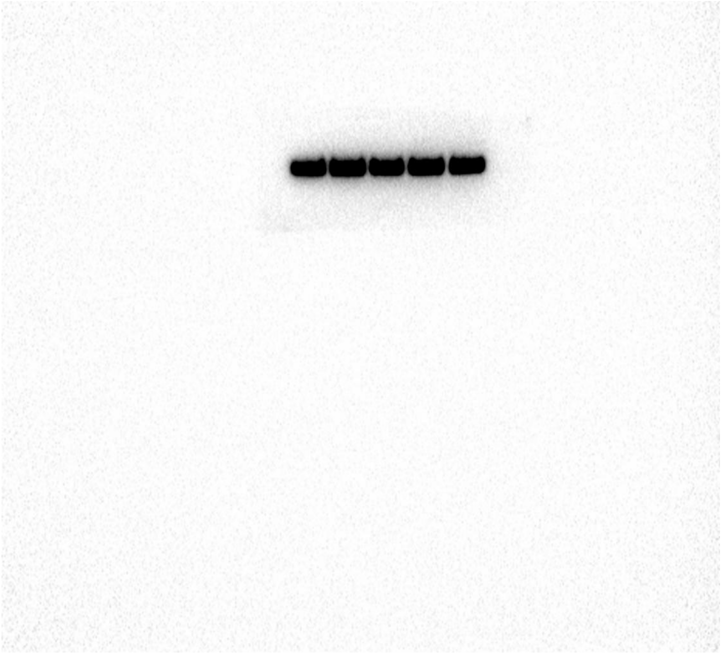

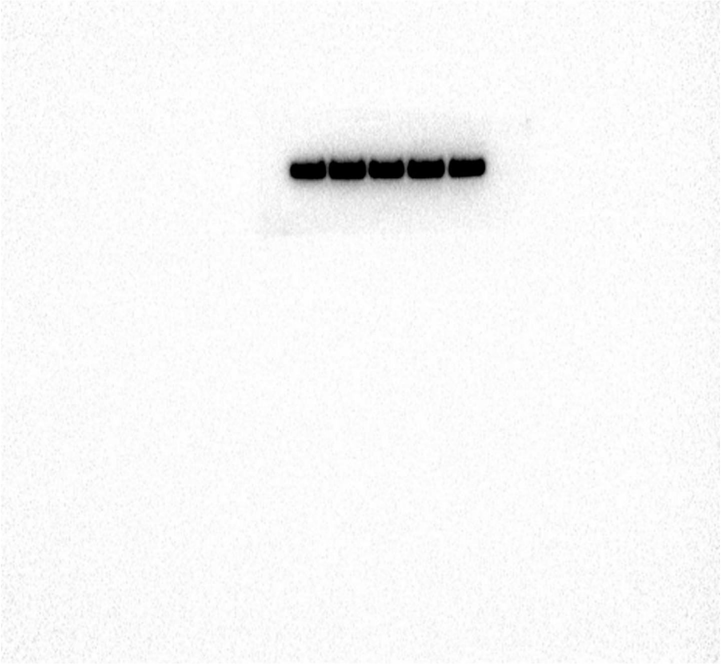

Original multiple exposure GAPDH-2 images

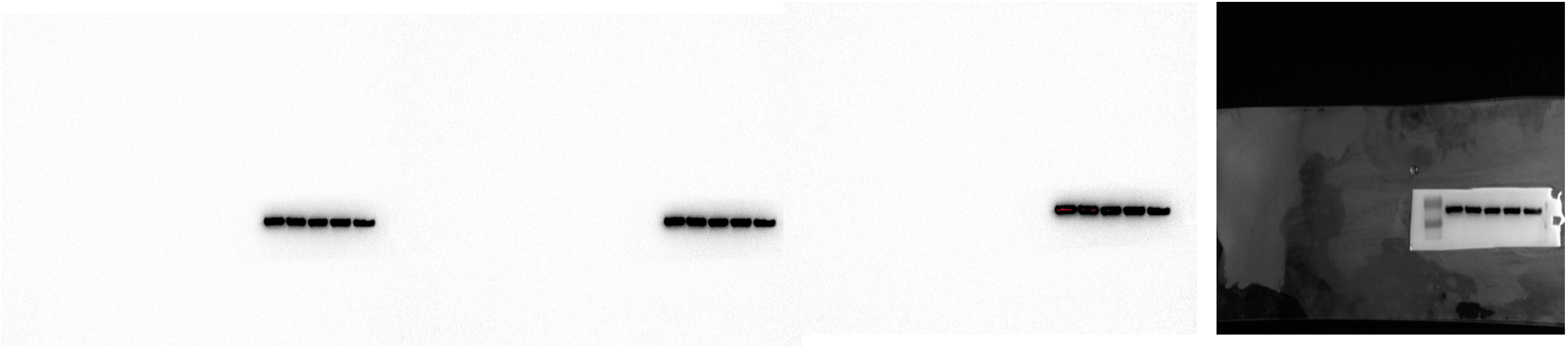

Original multiple exposure IL-1β images

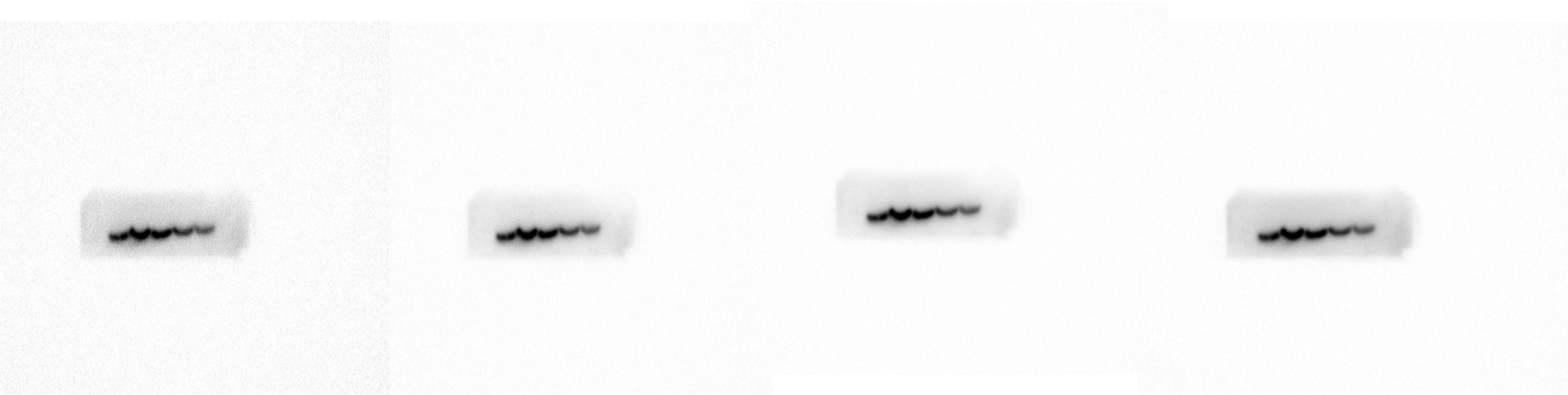

Original multiple exposure TLR-4 images

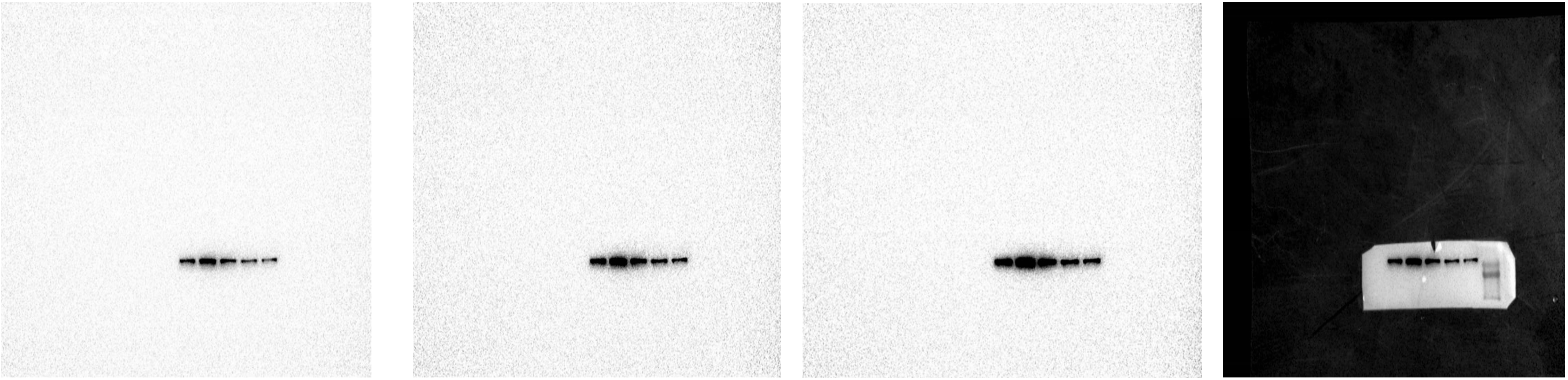

Supplement: Supplementary file 1 — Additional file 1: Figure S1. The original, unprocessed gel images. RAW264.7 cells were pretreated with piperine at 20–80 μM for 12 h and then stimulated by LPS (1 μg/ml) for 24 h. The protein levels of CD11c, IL-1β and TLR-4 were tested by Western Blotting and the original multiple exposure images are included in the Fig. S1. [file 12902_2020_617_MOESM1_ESM.pdf]
